# Supplementary material for: Evolutionary routes to biochemical innovation revealed by integrative analysis of a plant-defense related specialized metabolic pathway
Source: eLife. 2017 Aug 30;6:e28468. doi: 10.7554/eLife.28468 (PMC5595436; doi:10.7554/eLife.28468)
Supplement: Figure 2—source data 1. — This data was used to infer Figure 2A. [file elife-28468-fig2-data1.docx]

***Figure 2 - Source Data 1: NMR chemical shifts for four acylsugars purified from Salpiglossis plants***

|  |  |  |  |
| --- | --- | --- | --- |

**S4:19**(2,5,6,6) **S4:21** (5,5,5,6) **S5:21**(2,2,5,6,6) **S6:25**(2,2,5,5,5,6)

| **Carbon # (group)** | **^1^H (δ, ppm)** | **^13^C (δ, ppm)** | **^1^H (δ, ppm)** | **^13^C (δ, ppm)** | **^1^H (δ, ppm)** | **^13^C (δ, ppm)** | **^1^H (δ, ppm)** | **^13^C (δ, ppm)** |
| --- | --- | --- | --- | --- | --- | --- | --- | --- |
| 1 (CH) | 5.64 (d, *J* = 3.7 Hz) | 89.8 | 5.87 (d, *J* = 4.0 Hz) | 88.8 | 5.62 (d, *J* = 3.6 Hz) | 89.5 | 5.70 (d, *J* = 3.6 Hz) | 89.4 |
| 2 (CH) | 4.87 (dd, *J* = 10.4, 3.7 Hz) | 70.5 | 4.78 (dd, *J* = 10.3, 4.1 Hz) | 71.1 | 4.90 (dd, *J* = 10.4, 3.7 Hz) | 70.5 | 4.87 (dd, *J* = 10.4, 3.7 Hz) | 70.4 |
| -1(CO) |  | 172.8 |  | 177.6 |  | 172.3^d^ |  | 175.9 |
| -2(CH_2_) | 2.32 (dd, *J* = 15.7, 5.7 Hz), 2.06 (dd, *J* = 15.7, 8.3 Hz) | 41.0 | 2.41 (sextet, *J* = 7.0 Hz) | 40.8 | 2.30 (dd, *J* = 15.5, 5.8 Hz), 2.05 (dd, *J* = 15.5, 8.3 Hz) | 41.0 | 2.40 (sextet, *J* = 7.0 Hz) | 40.7 |
| -3(CH) | 1.82 (m) | 31.6 | 1.14 (d, *J* = 7.0 Hz) |  | 1.82 (m) | 31.6 | 1.12 (d, *J* = 7.0 Hz) | 16.1 |
| -3(CH_3_) |  |  |  | 16.2 |  |  |  |  |
| -4(CH_3_) | 0.88 (d, *J* = 6.7 Hz) | 19.4 | 1.64 (m), 1.45 (m) | 26.7 | 0.88 (d, *J* = 6.7 Hz) | 19.3 | 1.63 (m), 1.44 (m) | 26.8 |
| -5(CH_2_) | 1.34 (m), 1.20 (m) | 29.3 | 0.87 (t, *J* = 7.4 Hz) | 11.6 | 1.33 (m), 1.20 (m) | 29.4 | 0.86 (t, *J* = 7.4 Hz) | 11.7 |
| -6(CH_3_) | 0.88 (t, *J* = 7.4 Hz) | 11.4^a^ | 5.58 (dd, *J* = 10.6, 9.3 Hz) |  | 0.87 (t, *J* = 7.4 Hz) | 11.4^d^ | 5.57 (dd, *J* = 10.7, 9.2 Hz) | 68.9 |
| 3(CH) | 5.53 (dd, *J* = 10.7, 9.1 Hz) | 68.9 |  | 68.8 | 5.55 (dd, *J* = 10.7, 9.2 Hz) | 69.0 |  | 172.2 |
| -1(CO) |  | 172.3^b^ | 2.25 (dd, *J* = 15.8, 5.4 Hz), 2.02 (dd, *J* = 15.8, 8.5 Hz) | 172.2 |  | 172.3^d^ | 2.24 (dd, *J* = 15.8, 5.4 Hz), 2.01 (dd, *J* = 15.8, 8.6 Hz) | 41.1^g^ |
| -2(CH_2_) | 2.23 (dd, *J* = 15.5, 5.6 Hz), 2.02 (dd, *J* = 15.5, 8.4 Hz) | 41.2 | 1.79 (m) | 41.1^c^ | 2.23 (dd, *J* = 15.6, 5.5 Hz), 2.02 (dd, *J* = 15.5, 8.4 Hz) | 41.3 | 1.78 (m) | 31.5 |
| -3(CH) | 1.79 (m) | 31.6 | 0.89 (d, *J* = 6.7 Hz) | 31.5 | 1.79 (m) | 31.7 | 0.88 (d, *J* = 6.7 Hz) | 19.5 |
| -4(CH_3_) | 0.88 (d, *J* = 6.7 Hz) | 19.4 | 1.31 (m), 1.19 (m) | 19.5 | 0.87 (d, *J* = 6.7 Hz) | 19.4 | 1.30 (m), 1.18 (m) | 29.4 |
| -5(CH_2_) | 1.31 (m), 1.18 (m) | 29.4 | 0.86 (t, *J* = 7.4 Hz) | 29.4 | 1.30 (m), 1.17 (m) | 29.4 | 0.85 (t, *J* = 7.4 Hz) | 11.4 |
| -6(CH_3_) | 0.85 (t, *J* = 7.4 Hz) | 11.4^a^ | 4.93 (dd, *J* = 10.7, 9.3 Hz) | 11.4 | 0.85 (t, *J* = 7.4 Hz) | 11.4^e^ | 4.97 (dd, *J* = 10.3, 9.2 Hz) | 69.0 |
| 4(CH) | 4.92 (dd, *J* = 10.7, 9.2 Hz) | 68.6 |  | 68.6 | 4.97 (dd, *J* = 10.5, 9.2 Hz) | 68.9 |  | 176.5 |
| -1(CO) |  | 176.1 | 2.36 (sextet, *J* = 7.0 Hz) | 176.2 |  | 176.4 | 2.36 (sextet, *J* = 7.0 Hz) | 41.1^g^ |
| -2(CH) | 2.36 (sextet, *J* = 7.0 Hz) | 41.1 | 1.10 (d, *J* = 7.0 Hz) | 41.1^c^ | 2.35 (sextet, *J* = 7.0 Hz) | 41.1 | 1.10 (d, *J* = 7.0 Hz) | 16.5 |
| -3(CH_3_) | 1.10 (d, *J* = 7.0 Hz) | 16.5 | 1.66 (m), 1.44 (m) | 16.4 | 1.10 (d, *J* = 7.0 Hz) | 16.5 | 1.66 (m), 1.45 (m) | 26.7 |
| -4(CH_2_) | 1.67 (m), 1.44 (m) | 26.6 | 0.90 (t, *J* = 7.4 Hz) | 26.6 | 1.66 (m), 1.44 (m) | 26.7 | 0.90 (t, *J* = 7.4 Hz) | 11.8 |
| -5(CH_3_) | 0.90 (t, *J* = 7.4 Hz) | 11.8 | 4.15 (ddd, *J* = 10.6, 6.2, 2.5 Hz) | 11.8 | 0.90 (t, *J* = 7.4 Hz) | 11.8 | 4.07 (ddd, *J* = 10.2, 5.1, 2.2 Hz) | 71.2 |
| 5(CH) | 4.14 (ddd, *J* = 10.2, 5.5, 2.8 Hz) | 71.9 | 3.63 (dd, *J* = 12.4, 2.4 Hz), 3.60 (dd, *J* = 12.4, 6.5 Hz) | 61.5 | 4.05 (ddd, *J* = 10.3, 5.2, 2.1 Hz) | 71.1 | 3.66 (dd, *J* = 12.8, 2.3 Hz), 3.59 (dd, *J* = 12.8, 5.1 Hz) | 61.7 |
| 6 (CH_2_) | 3.61 (m) | 61.9 | 5.87 (d, *J* = 4.0 Hz) | 88.8 | 3.66 (dd, *J* = 12.8, 2.3 Hz), 3.58 (dd, *J* = 12.8, 5.3 Hz) | 61.6 | 5.70 (d, *J* = 3.6 Hz) | 89.4 |
| 1′(CH_2_) | 3.66 (d, *J* = 12.3 Hz), 3.54 (d, *J* = 12.3 Hz) | 64.6 | 4.13 (d, *J* = 11.6 Hz), 4.06 (d, *J* = 11.6 Hz) | 64.1 | 3.63 (d, *J* = 12.3 Hz), 3.55 (d, *J* = 12.3 Hz) | 64.3^f^ | 4.14 (d, *J* = 11.8 Hz), 4.04 (d, *J* = 11.8 Hz) | 64.1 |
| -1(CO) |  |  |  | 175.9 |  |  |  | 175.8 |
| -2(CH) |  |  | 2.41 (sextet, *J* = 7.0 Hz) | 41.1^c^ |  |  | 2.44 (sextet, *J* = 7.0 Hz) | 41.1^g^ |
| -3(CH_3_) |  |  | 1.15 (d, *J* = 7.0 Hz) | 16.7 |  |  | 1.17 (d, *J* = 7.0 Hz) | 16.7 |
| -4(CH_2_) |  |  | 1.68 (m), 1.49 (m) | 26.9 |  |  | 1.70 (m), 1.51 (m) | 26.9 |
| -5(CH_3_) |  |  | 0.91 (t, *J* = 7.4 Hz) | 11.7 |  |  | 0.93 (t, *J* = 7.4 Hz) | 11.7 |
| 2′(C) |  | 104.2 |  | 103.7 |  | 104.7 |  | 103.0 |
| 3′(CH) | 5.17 (d, *J* = 7.8 Hz) | 80.1 | 4.17 (d, *J* = 8.9 Hz) | 78.4 | 5.20 (d, *J* = 7.6 Hz) | 79.3 | 5.23 (d, *J* = 8.1 Hz) | 79.0 |
| -1(CO) |  | 172.3^b^ | 4.31 (t, *J* = 8.9 Hz) |  |  | 172.0 |  | 171.5 |
| -2(CH_3_) | 2.26 (s) | 21.0 | 3.71 (dt, *J* = 9.2, 2.3 Hz) |  | 2.22 (s) | 21.0 | 2.20 (s) | 20.9 |
| 4′(CH) | 4.60 (t, *J* = 7.8 Hz) | 71.5 | 3.88 (dd, *J* = 13.4, 2.4 Hz), 3.71 (dd, *J* = 13.4, 2.3 Hz) | 72.6 | 4.35 (t, *J* = 7.6 Hz) | 73.9 | 4.35 (t, *J* = 8.1 Hz) | 73.5 |
| 5′(CH) | 3.89 (m) | 82.4 | 4.13 (d, *J* = 11.6 Hz), 4.06 (d, *J* = 11.6 Hz) | 81.0 | 4.08 (ddd, *J* = 9.6, 6.1, 3.6 Hz) | 80.4 | 4.04 (ddd, *J* = 8.2, 5.7, 3.6 Hz) | 80.2 |
| 6′(CH_2_) | 3.89 (m), 3.71 (m) | 59.9 |  | 59.7 | 4.41 (dd, *J* = 12.1, 6.2 Hz), 4.28 (dd, *J* = 12.1, 3.6 Hz) | 64.3^f^ | 4.40 (dd, *J* = 12.2, 5.6 Hz), 4.30 (dd, *J* = 12.2, 3.5 Hz) | 63.8 |
| -1(CO) |  |  |  |  |  | 171.7 |  | 171.7 |
| -2(CH_3_) |  |  |  |  | 2.12 (s) | 21.0 | 2.13 (s) | 21.0 |

^a^Two ^13^C signals not resolved in 2D spectra (11.37, 11.39 ppm)

^b^Two ^13^C signals not resolved in 2D spectra (172.27, 172.29 ppm)

^c^Three ^13^C signals not resolved in 2D spectra (41.08, 41.10, 41.13 ppm)

^d^Two ^13^C signals not resolved in 2D spectra (172.34, 172.35 ppm)

^e^Two ^13^C signals overlapping

^f^Two ^13^C signals overlapping

^g^Three ^13^C signals not resolved in 2D spectra (41.10, 41.12, 41.14 ppm)
