# Supplementary material for: Evolutionary routes to biochemical innovation revealed by integrative analysis of a plant-defense related specialized metabolic pathway
Source: eLife. 2017 Aug 30;6:e28468. doi: 10.7554/eLife.28468 (PMC5595436; doi:10.7554/eLife.28468)
Supplement: Supplementary file 2. — The transcript identifier as per the assembler Trinity nomenclature is noted in the part after the bar (|). Green and yellow highlighted sequences are the two regions targeted for VIGS in Salpiglossis. [file elife-28468-supp2.docx]

***Supplementary File 2***

Sequences identified in this study. The transcript identifier as per the assembler Trinity nomenclature is noted in the part after the bar (|). Green and yellow highlighted sequences are the two regions targeted for VIGS in Salpiglossis.

>SsASAT1|c40486_g1

ATGGTTGCCTCAGCACTAGTTTCTTTGTCGAAGAAAATTATCAGACCTTTCTCTCCAACCCCTTTTTCCGAAAGAATTTACAAGCTTTCCTTTATTGATCAATTCAATAGTACACAATATATCCCCTTAGTCTTCTTCTATCCCAAGAACAAGGGAAATATTGAACCAAGTGATATGTGTAAGGTTTTTGAGAATTCCCTCTCAAAAACCTTAGCTGCTTATTACCCTTTTGCAGGAACATTAAGGGACAATATATGTGTTGAGTGTAACGATATAGGTGCTGATTTCTTCAAGGCTCGTTTCGATTGTCCAATGTCTGAAATTCTCAAAAGTCATAATAGAGATGTCAAAGAAAGTGTATATCCCAAGGATATACCATGGAGTGATGCATCAAACAAAAAGTTGGTCACGGTTCAATTTAACCAATTTGATTGTGGAGGAATAGCTCTAAGTGCATGTATATCGCACAAGATTGGAGACTTTTGTACGGTGATTAACTTTTTTCACGATTGGGCTGCAATTGCTCGTGATTCTAATGGAAAAGTATGTCCTCAATTTATTGGATCATCCATCTTCCCACCTACCAATGAACCTGTGAATGAGCCTCCTCGTATAAAATCATGTGTAACGAAAAAGTTAGTTTTTTCAAACCATACATTAAAATCTTTCATTACTGAATCATCATCACTAGAAGTTAAAGAACCAACTCGGGTAGAAATACTCACATCACTTCTTTATAAATGTGGTATGAAAGCGGTATTGGAAAATAATTCTTCTAGTTCTTCAATTTTCAACCCGTCTATTCTGTTCCAAACCGTGAATTTAAGGCCTTTTATACCTTTGCCAGAGAATACTGCTGGAAATTTTAGTTCTTCCCTTTTTGTACCGACATATATTGAAGAAGAAACAAAGTTATCTACATTGGTTAGTCAACTAAGAAAGGGAAAAGAAGAGGTCATTAATAACTTCAAGAAATGTGAAGGGGGTCAAGATTTGGTGTCAGCAACAAAAGGACCATTTCAAGAAATAAGAAAGTTGTACAAGGAGGTGGATTTTCAACTGTATAGGTGTAGTAGTTTGGCTAATTATCCAATATATGATGTAGACTTTGGATGGGGTAGGCCAAAACAAATAACTATGGCGGATTCTCCATTGAGAAATACTTTCTCTTTGTTTGACGACAACACTGGGAATTATATAGAAGCACTGGTAGCTTTGGATGATGAAATTACTATGTCTACGTTTGAGAGAGAGATGGAGCAACTTCTTGAATCTCAACTTCCAACTGAAGAAAATAAAACGGAAGCTAGCGCTCATTGTTGA

>SsASAT2|c49208_g1

ATGGCTGCTTCAAGGCTTGTTTTGCTTTCCAAAAAGATGATTAAGCCTTCTTCTCCAACTCCAATTTCACATAGAATTCACAAGCTCTCACTTATGGACCAAATGGGAACTCACTCCTACGTTCCGTTTTCGTTCTTTTACCCGAAACAAGACGCTGCAAGCTCGCTCGAACCAACAAAGGTATCTCGAATTCTTGAAAAATCCCTTTCGAAAGTTCTAACATCATATTATCCGTTAGCAGGGCGCGTAAGAGACAATTCATTTGTCGAATGTAATGACATGGGAGTCGATTTCTCTCAAGTCCAAATAGATTGTCCAATGTCCAGAATTTTCGATGTCCCTCGTGCTGAGATGGACAATTTAATATTTCCTAAAGATCCTTGGATCCCGTCTACGGACAGTTTAGTCGTAGCACAACTCAACCATTTCGAATGTGGTGGCGTTGTACTAAGCACATGCATTTCCCACAAGGTTGCCGACGGATACAGTATCGCGAAATTCCTCAGGGACTGGGCTATTGTCGCGCGTGATTCAGGAGAGAAACCATCTCCCCTGTTTAATGGAGCGTCGATACTTAAACCAACCAACAATTCTTCAACTCAAGTGGTTGATCCCTCTTTATATAAAACAACATCGAAAAGGTACCATTTTTCTGCCTCCAAGTTAAAAGCTCTTAAGGCCATGATTTCAGCTGATTCTGGAAACCAGATTGTTCCTACAGCCGTGGAAGCCGTTACTGCGTTGCTATTCAAAAGCATCAATGCACGAAATTTCAAGCCATCGATGTTGATGCAAACGGTAAATCTACGTGGAAAAAACAACGACGCGCTACTTCCAGCAGACTTGGTCGGGAACGTTATTTTTCCTTTCGTCGTATCAGCAGCGAATGAGGACGAGCTGAATTTGCAAAGACTCGTTAGCGAGCTTAGAGAAGGAAAAGAGAAGATACACAATACGCTTGAATGTATTAAATCGGAAGAGTTACTCTGTTCAAAGGTATGTGAAATAGCTAGAGAGATAAACAAGCGAACCACGAGGGATGATTTTTCTATGTACAGATTTACTAGTGTAAGGAAATTCCCTTTGAACGATATCGATTTCGGATGGGGAAGGCCAAGAAGAGCGGATTTTGCTACTGCGCCGATCAATCTGATTTTCTTGATGGATGATCAAAGTGGGGATGGAGTTGAAGTGCTATTAAACTTGGGAGAAGAAGAGATGTCTACATTTCAAAGTAATGATGAGCTTCTTCAATTTGCTTCTCCGAGCTGA

>SsASAT3|c36223_g2

ATGGGTGGTGTATCAAACCTTGTGTCATCAAGTTGCAAAAAAATAATCAAACCTTACTTTCCTACTCCAACTTCACTTCGTTGTTGCAAGCTCTCTTACCTCGATCAAATAGTCGGTCGCATTTATGCGCCTATGGCCTTTTTTTACCCTAAGCGTTCCGATATTATTAAACCAAGTGTTAATATATCCCAACATCTGCAAAATTCCCTTTCTAAAGTTTTGACACATTATTATCCATATGCGGGAAAGTTGAATGACAATGTCTCTGTTGATTGTAATGACAGTGGAGTAGAGTTCTTCACTACCAAAATTAATTGTCCAATGTCTGAAATCGTCAACAACCCTTATGCTGATAAACAAGATTTAGTCTTTCCCAAAGGAATTGCTTGTACACCTTCATACGAGGGTAGCCTTGCAGTTATTCAACTTAGTCATTTTGATTGCGGAGGATTTGCAATCAGTGCATGTGTGACACACAAAATTGGAGATGCATATACAATAGGCAATTTCATTAACGATTGGGCCACTATAACACGTGACCCAAGTTCAAAAATATTACCCGGTCCTCAATTTAACGGAGCCTCTTTTTTCCCACCTTCAAACGACCTACCAAATGAATCCAATTTTATTGTGCCAAAAAGTAAAGAGTGTTTATCCAGAAGTTTTGATTTCTCCCAATCTAAATTAGCTTCTTTAAAATCCAGGGTCATAAATAGTTTACCAGAGGTAAAAAATCTAACTGACACTGAGATTGTGTCAGCATTTATTTACCAACGAGCCATGGCTACAAAAAATGCTGTTGTAGGCTCAATTCAACCATCGTTACTCATCCAAGCTGCAAACTTGAGGCCACCAGTGCCAAAAAGCACTATGGGAAACATTGGTTCCTTCTTTTCTGTACAAACAACAGAAGAGAATGAAATTGATCTACCAAGTCTTGTTGGTAAACTACGAAAGGCGAAAGAAGAATATCGACAAAAATACAAACATGCTAAAACAAATGAACTTTTACCTATAACAATAGGACTATATAGAGATGCAGATGAATTTTTGGTAAACAATTGTTATGACATTTATAGGTTTAGCAGTATTAAAAAATTTCCCTTGTATGATGTAGACTTTGGATGGGGAAAGCCTGAGAGGGTTAGTCTACCAAGTAATTGTCCAGTTAGAAACTTCTTTGCCTTGGTTGATAATAAAGCTAGGGATGGAATGGAAGTAATAATGTACATGAAGGAACAAGATATTTTGGCACTCGACAAGGATGAAGAGCTCCTAGAGTTTGCTTCTCCAAGCACTTAA

>SsASAT5|c48744_g1

ATGGAACTAATCTCCCAAGAATTCATCAAACCTGTTTCTCCAACCCCCGATCACCTCAAATTCTATGAGTATTCCATTTTGGATCAGCTCATTGGTCCATCCCTTTACACCCCTGTACTTCTATACTATCCATCTCCACCAGATAATAATAATAACAAAAAACCGGAGGCTATCAATACATCAAGATTAAAACACTTGAAGAAATCCTTGTCACAAATTCTTGCTCACTATTACCCTTTGGCAGGAAGACTTCGAAACGACAACACTGGCGTCGATTGCAACGATGAAGGCGTTCCATTTCTCGAGGCATTTGTGCACAATCATCGCTTGCAAGACATTCTCGATGGAAAAAGAGTAGTTACAGAATCTTTGGTTCCACTCACCAATTACGAGTCAGTATTTCCATCAAACACGTTGCTTCTTGTTCAAGTTACCTTGTTTGAATGTGGAGGAATGGCTGTTGGTATTTCAGCTACACATAAAATATTAGATGCTCGTTCTTTGATCACCTTCTTAACTGATTGGGCAGCATTGACGCGCCAAGACGATCCCAATTTTACACTCACGCAACTTGTCCCCCTTTCCAAAATTATACCACCAGCTAATGGATTGCCACCCTCAATTCCGGTTGAAGGCTTTATCTCAACGGAGCCATGTGTTAGGAATATTTTTGTATTCAGACCTTGTAGCATTGCTGATCTGAAGATTAAGGCAGCCAGCGAGAACATACTGAGGCCTTCGCGCGTTGAAGTAGTGACAAGTACTATCTGGATGTGCCTAATGGAAAACTCCAAAAGGCCGTGTTTGATAACACACATGGTAAACTTGAGGAAGCGAGTTGACCCTCCAATGCATGATCACCATCTTGGAAACTTCATTGGGATGGTTGTAGCACATAATGATGAAAATCATATAGCTAATATGGTGGCTTCATTACGAAAAGGGATTTCCGAGTTTGAAAAGAAGTGTCTGAAGGGGGAACGGGAGGCCTTGGGGGCTAGCATAGTAAACCACGCAACAGAATCAATAAATTACTTGGTTAGCAGAAAGGATGCAGATTTGTACAAATTCAATAGCTGGTGCGGTTTTCCATTTTATGATGTGGATTTTGGGTGGGGAAAGCCAGTTTTGTCTAGCACAGCTGAAGGGAAATCCAAGAATTCTATAAAATTGATAGATACAAAGGATGGTGGAGTGGAAGCTCTTGTTTGTCTGAGTGAAGAAGATATGAAAGTGTTTGAGAAGAATACAGAGCTGCTAACTTATGCTACTCTGAAACCCAATTCGCATGTGTAG

>SsPDS|c55490_g2

ATGCCCCAAATTGGACTTGTTTCTGCTGTTACCTTGAGGGTTCAAGGTAATTCACTCTATCTTTGGAGTTCGAGGTCTGCTACTGAAAGTCACGTTGGTCGCGCACAAAGGAATTCGTTATGTTTTGGTGGTAGCGACTCCATGGGCCATAAATTAAAAATTCATACTCCCCATGCCACGGCTAGAAGATTGGCAAATGGCTTCCATCCTTTAAAGGTAGTTTGCATTGATTATCCAAGACCAGAGCTAGACAATACAGTTAACTATTTGGAGGCTGCATTGTTATCATCATCATTTCGTACATCTCCTCGCCCAACCAAACCATTGGAGGTTGTTATTGCTGGTGCAGGTTTGGGTGGTTTGTCTACAGCAAAATATTTGGCAGATGCTGGTCACAAGCCGATATTGCTCGAGGCAAGAGATGTCCTAGGTGGAAAGGTAGCTGCATGGAAAGATGACGATGGAGATTGGTATGAGACTGGGTTGCACATATTCTGTAAGTTTAACTCCTCAATAATACTGTCATTGCAATTTCTTTTGAGATCTTTTTTGTCCATTAGACAGATAGTTATCCCTGTTTGTCTTTTGTCTTTGCAAATACCAATTATTGTCAGTCAATATGTATTATACATTGCTTCTCATTTTCATCTGTTAATTTCCTGTCGTGACTCATACAAGTTGGTACTTCATCTCTTTTAAGTTGGGGCTTACCCAAATATTCAGAACCTGTTTGGAGAATTAGGGATTAATGATCGATTACAGTGGAAGGAACATTCAATGATATTTGCAATGCCTAATAAGCCAGGGGAATTCAGCCGCTTTGATTTTCCCGAAGCTTTGCCTGCACCATTAAATGGAATTTTGGCCATCCTAAAGAACAATGAAATGCTAACATGGCCAGAGAAAGTCAAATTTGCAATTGGACTCTTGCCGGCAATGCTTGGAGGGCAATCTTATGTGGAAGCTCAAGACGGGTTAAGTGTTAAGGATTGGATGAGAAAGCAAGGTGTGCCTGATAGGGTGACAGATGAGGTGTTCATTGCCATGTCAAAGGCACTTAACTTCATAAATCCTGACGAGCTATCGATGCAGTGCATCTTGATCGCTTTGAACAGATTCCTTCAGGAGAAACATGGTTCAAAAATGGCCTTCTTAGATGGTAATCCTCCTGAGAGACTTTGCAAGCCAATTGTTGAACATATCGAGTCAAAAGGTGGCCAAGTCAGACTAAACTCACGAATAAAAAAGATTGAGCTAAATGAGGATGGAAGTGTGAAGTGTTTTATACTGAACAATGGTAGTACAATTGAAGGAGATGCATTTGTGTTTGCAACTCCAGTGGATATCTTCAAGCTTCTATTGCCTGAAGAGTGGAAAGGGATCCCATATTTCCAAAAGTTGGAGAAGTTAGTCGGAGTTCCTGTGATTAATGTCCATATATGGTTTGACAGAAAATTGAAGAACACGTCTGATAATCTGCTCTTCAGCAGAAGCCCACTACTCAGTGTGTACGCTGACATGTCTGTCACATGTAAGGAATACTACAATCCCAATCAGTCGATGTTGGAATTGGTTTTTGCACCAGCAGAAGAATGGATATCTCGCAGTGACTCAGAAATTATTGATGCTACGATGAAGGAACTAGCAAAACTTTTTCCTGATGAAATTTCAGCAGATCAGAGCAAAGCAAAAATATTGAAGTATCATGTTGTCAAAACTCCAAGGTCTGTTTATAAAACAGTTCCAGGTTGTGAACCCTGTCGGCCGTTGCAAAGATCCCCTATAGAGGGTTTTTACTTAGCCGGTGACTACACGAAACAGAAATACTTGGCTTCAATGGAAGGTGCTGTCTTATCAGGAAAGCTTTGTACGCAAGCTATTGTACAGGATTACGAGTTACTTGATGCCCGGGGCCAGAGGAAGCTGGCAGAAGCAAGCCTAGTTTAG – 3’UTR – CGGAGTGGAGCTACAATTAGTGTTTGTACACAGCATATATACAAGAGAACCAAATACACAGTGTTACATAATTGAAGGGGCAAGCTCCTACCACAAACGTCAAAAAAAGATGCTTCAAGCTTAACCTTCTTTAATCAGAAATTGAAATGTAAGCACATACCATGTTATTTAATCAGAAATTGAAATGTAAGC

>SS_c52698_g1

ATGGTGTCTTCAAAACCCGAAGCTGGTCTAATCTACAACATCAAATTATCCTCAGTTGGACCAGCAAGGGTAACAGGACAAGATGTGGTTTATGAGCCAAGCAACATGGATTTAGCCATGAAACTACATTATTTAAGAGGGATTTATTATTTTGAAAACCAAGCATTTCAAGATTTCAACATATATAAAATTAAAGAGCCAATGTTTTCTTGGTTGAATCATTTTTATATGACATGTGGCAGGCTTAGAAGGGCAGAATCAGGGGGGCCTTATATAAAATGCAATGATTGTGGAGTTAGGTTTATTGAAGCACAATGTGATAAAACTTTGGATGAATGGCTTGAAATGAAAAATTATAATAATTCTCTTGAGAAGTTACTTGTTTCTAATCAAGTTCTTGGTCCTGAATTGGCTTTCTCCCCTCTTGTCCTCATACAGCATACTAAATTCAAATGTGGTGGAATTTCATTGGGCTTAAGTTGGGCTCATGTACTTGGAGACATATTCTCAGCAGCTGAATTTATGAACCTACTGGGAGAAGTGGTTAGTGGTTACAAACCAACCCGGCCCATTAACTTGGCCCATTCATTGACCAAAGCAAATTCAACCCAAATCCTACAAAAGATTGTGGAGGATCCAATTTCCATAAGACGGGTCGACCCGGTTGAAGATCATTGGATTGTTAAAAATAGTTGCAAGATGGAGCTATTTTCATTCCATGTCACTGCCTCCAAATTGGGCCAGTTGCAATCAAGAGTGGGCCATCAAGGCCCATTTGAATCGCTATGTGCAGTTATCTGGCAGTCCATTTCAAGAATTAGAGATGGGCCTGAGCCACAAGTTGTGACCATTTGTAAAAAAGGTGAGGGAAAAAAAGAGGGCCTTGTGGGAAACACTCAAATCATTGGTGCACTAAAGTTGGAATATTCAATTAGAGAAGCCAATCCTAGTGAACTAGCAAGGTTAGTTAAAAATGAGATCATCGACGAGCGATTAAAAATAGACGAAGCTGTGGAAAAAGAACATGGAGTATCGGATCTTGTCGTTTATGGAGCAAATTTGACTTTTGTGAACTTAGAAAGTGTTGATTTCTATGGACTTGATTGGAAGGGACACAAGCCAATGAATGTAAGTTACATAATCGACGGAGTTGGTGATGCTGGGACCGCTATGGTGTTTCGGGGGCCCAATGATATTACCAAGGAGGGTGATGAAGGAAGAACTGTGACAATGATTTTGCCCGAGGATGAGATAATGGCATTGAAAGTTGAGCTAAACAAAGAATGGTCTATTGCTTGTATTTAA

>SS_c49704_g1

ATGTCAAATTGCAATGGAAATGGAGTTTCACATTTTGGCTCCAATAACTTGCAAATTGAAGCAATCCAAACAGTGATACCAATAAAGCCAACTAAGCCAAGGTTGTCCCGGCGAATCGCCGTGGCTGATCAAAATGGAAATTTTCTCCAGAGGCGTTTTCACGCGGTCCTTTGCTATAACAAGGCCTCAGAGGAGGATTCAGGGTGGATCGTTGCTGGTCGGATCAAAGAGTCACTTGGAAGGGCACTTGTTGAAAATCCATTACTTGCTGGTAGACTTAAAAAAGGAGAAAATGGGGATTGTGGAGAGTTTGAAATTGTATCGAATGATTCTGGTGTTAGATTGGTTGAGGCTATAATGCCAATGAAGTTGGCAGATTTTCTTGATCTCAAGGATAAGAAAATTGCAGAAGCTGAGCTTGTCTTTTGGGAGGATGTTCATGAACCAAATCCTCAGTTTTCTCCTCTTTTTTATGTCCAGGTGACAAATTTCAAGTGTGGAAGATACTCAATTGGGATAAGTTGCAGCCTTTTTCTAGCAGATCCTTATGCCATGACTAGTTTCTTGAATAAATGGTTCAAAATTCACAATAATATGGTATCAGAATCAGACACACCCAAAATTCCAACATTTTTCCTTCCAAGTTTTAGGAAAATCGGTTGTTCACCAACTTTATCAATGAGCTCCACTACATGCCACCAAGTTAACGAAACGCTAATCTTTAAAATACCCGCGAAGATCTTGAATTTAAAAGATGACATGCACTTGAATCTTGCAGCAAAATGTGTTGGGGAAGCAGAAGACAAACTTGGTAAGAAGTCGTCGTCAAAATTCTGTTTGTTTGTTAAAGATACCTCGGAGGATGTTAAAGTGGAAACTTATTCTCGAGAAGGGATTTTGCCAGAAATATTTGGTTCTATCAATAGTGGATTAGTTTCAACCAGCTTCGATGATTTGGAAGCTGATAACATAAGGTTTAATGAAGAAAATAAGGCTGTCTTCTTTTCATGTTGGATTAACTCAGGAAATGATGATGATCTTGTGCTGATTACTCCATCTGTTGGTGAAGGTGACTCTGAAATGAAGGTTATAGTAACTGTTAGATATTAA

>SS_c49383_g1

ATGGGATCATTGGTTGCCCTCTTTTGTTAGTTCAGGTGACCCGTTTTAGATGTGGGGGATTTAGTGTTGGGTTCAGACTTAATCACACAATGATGGATGTATATGGTATGAAATTGTTTTTAAACGCGTTAACCGAATTAATGGGAGGAGCTGTTACACCTTCTATATTACCTGTATGGAAAAGGGATCTCCTAAGTGCTAGATCATCACCACGCATTACATGCACACACCATGAGTTTGATGAGTACTCCTCAAGGTATAATAATACAATTGCATGGTTAGACAAGAAGTTGGTCCAACAGTCTTTCTTTTTTGGAGACGAAGAGGTGGAAGCCATTCGAAATCAGTTACTAGACGATTGTCCAAATAGTACTAGTACAAAATTCGAGTTAGTAGCTGCATTTTTATGGAAGTATCGTACAATTGCTCTTGCTCCGCATCCTGAAGAGATTGTTCATCTCACTTACCTTATCAATGCACGCGGAAAATCATCGCTAAACCAATTCCAACTACCACGCGGGTACTATGGGAATGCGTTCGTTTCTCCAGCAGCAACATCAGAAGCAGGTTTGCTATGTTCAAGTCCATTAGCATATGCACTTGAATTGGTTAAGAAAGTTAAAGATCACGCAAACGAAGAATACATTAGATCAGTGGCTGATTTGATGGTGATTAAAGGGCGACCTGAGTTGAAGCAATCTTGGAATTTTATTATCTCAGATAATAGATTTGTTGGATTTGATGAAGTTGATTTTGGATGGGGAAAGCCCATGCTCGGAGGGGTTCCAAAAGCTTTATCTTTTATCAGCCATTGTGTACCTCTTTCAGATAACAAGGGGGGAAAAGGTATTCTCATAGCGATAAGTTTGCCTCCACTGGCCATGGAAAAATTTCAAGCGATTGTCTACAAGGTGACTTCCAAAAAATTGTCCAAGGCATCCCCATAA

>SS_c57127_g2

ATGGCTGCCAAAGTGGAGATGATATCCAAAGAAATGATCAAACCATCAACCCCAACACCTCCTTCCCTTAGAACCCACAAATTATCACTTCTCGATCAAATTGCACCCCCGGTTTTTCTCCCTCTAATTTTCTTCTACCAATATGAAGTACTTGACAATAACGACCGCACCAGAAAGTTACAGTCCTTGAAAAATTCTCTATCCGATGCTTTAACCCGATTCTACCCATTAGCTGGAACACTCAATATTAATAATAGTACCGTTGATTGCAATGATACTGGAGCAGAGTTTATTGAAGCTCAAGTTCATGGTTACACCCTCTCACAAGTTATGGAAAATCCAAATATTGAGGAATTAGCACAATTCCTCCCAATTCATGAAGCTTGTGGCATAGGGAATCATGATGTCCTTTTGGCAATCAAAGTTAATTTGTTTGATTGTGAAGGGATCGCAATTGGTGTGTGCATGTCACACAAAGTTGGCGATGGCGTGTCCCTTGTGACATTCATCAATTCATGGGCAGCCATTGCCCGAGGTGACACTGAAATTGTGCAGCCTAATTTCAACTTGGCAAGTCTTTTCCCATCAGTAGACTCGTTTAATTCAATTCACAATTCATCCATAGGAATAACTAACGAAAAACTTCTGATAAAAAGATTTGTTTTCGATAAGGAAAAGATTGATGCTCTCAAGAAATCGACTTCTATGGCCTCAGGATCAGGAGTGGAGGATCCCACTCGTGTGGAAGCCCTCTCGGCCTTCCTATGGAAACATTTCATAGAGGCTTCGAAGTTGAAAATAGACTCCAAAAAAACGTTTGCTACAATTCATATAGTCAATATGAGGCCAAGAATGAACCCAACCTTACCAGACCACTTTTTTGGGAACCTTTGGACAGTTGCATTAGCATTAAACACAATTAACTCAGAAACAAATAAATTAATGAAAACTACTAGTGATGATGATTTGGTGTACCAGTTGAGAAGTGCAATAAGGAGAATCAATGGTGACTATATAAATATGGTACAAAATAAAGAAGAGTTTCTGAAACATATAGGTAAGTTAGTAGAGTTATTTTCAAAGGGAGAAGTTGAGTTTTCATGTTATTTTACGAGCTGGAGTAAGTTTCCAGTGTATGAAGTGGATTTTGGATGGGGAAAACCAAGCAGGGTATGCACTACCACTTTGCCTTACAAGAATATGATCTTCTTCTTGCCTACAAAATGTGGAGAGGGAATAGAGGCATATGTTAACATGCCCAATGAAGATGTCTTTTCCCAAGCTTTGACATAA

>Ss_c57127_g3

ATGGCTGCCAAAGTGGAAATGATATCCAAAGAAATGATCAAACCATCATTGCCAACACCTCCTTCCCTTAGAACCCACAAATTATCACTTCCTGATCAAATTGCACCCCCAGTTTTCATCCCTTTAATTTTCTTCTACCAATATGAAACAGTTGACAATATCGACCGTGCCAGAAAGTTACACTCCTTGAAAAATTCTCTATCTGATGCTTTAACTCGATTCTACCCATTGGCTGGAACACTTAATATTAATAGCAGTACCATTAATTGCAATGATATTGGAGTAGAGTTTATGGAAGCTCAAGTTCATGATTACAGCCTCTCACAAGTTATGGAAAATCCAAAAATTGAGGAATTAACACAATTTCTCCCAATTGAAGCTTGTGGCATGGAGAACCATAATGTCCTTTTAGCAATCAAAGTTAATTTTTTTGATTGTGGAGGGATTGCAATTGGGGTGTGCATGTCTCATAAAGTTGGTGATGGCTTGTCCATAGTAACATTCATCAATACATGGGCAGCCATAGCCCGGGGTGACACCGAAAAAATTGTGCAGCCAAATTTCAACTTGGCAAGTCTTTTCCCACCAGTAGACTTGTCTAGTTCAAGTTACAATTCATCTATAGGGATAACTAACAAAAAACTTATGATAAGAAGATCTGTTTTCGATAAGAAACAGATTGATGCTCTCAAGAAATCGGCTTCTATGGCCTCAGGATCACGAGTGGCGGATCCCACTCGTGTGGAAGCCGTCTCGACCTTCCTATGGAAACATCTCATAAAGACTTCAAAGTCGAAAATAAACTCCAACAAAATGTTTGCTGCAATTCATATAGTTAATATGAGGCCAAGAATGAGCCCAACCTTACCTGACCACTTTTTTGGGAACCTTTGGACAACAGCACTAGCATTAATCACAAATAATTCAGAAACTAGTGATGATGAGTTGGTGTACGAGCTGATAGGTGCAATAAGAAAAATCAACAGTGAATATATAAATATGTTACAAAATGGAGAAGAATTGTTGAAGCATGCGGGGAAGTTAGTTGAGTTATTATCAAAGGGAGAAGTTGAGTTTTGTTGTTTTACGAGCTGGTGTAGGTTTCCAGTGTATGAAGCGGACTTCGGATGGGGAAAACCAATAAGGGTATGCACTACCACTTTGCCTTACAAGAATTGTATCTTCCTCTTGCCTACAAAATGTGGAGAGGGAATAGAGGCATATGTTAACATGCTAAACCATGATGTCCTTTACGAGCTCTGA

>HnASAT1|HN_c58659_g1_i1

ATGGCTGCCTCAGCTTTACTTTCTTTATCCAAAAAAATCATAAAACCATTTTCTCCAACACCATTTTCTGAAAGAATTTACAAGCTTTCTTTCATTGATCAATTCAATACTACACAATATAACCCCCTCGCCTTCTTCTATCCCAAAAACAAGGGAGTACCCTCAATTGATCCAAATGATATGTGTAAGGTTATAGAGAATTCACTTTCAAAAGCCTTAGCTGCTTATTACCCTTTTGCTGGAACATTGCGAGACAATATTTATATCGAGTGTAACGATATAGGTGCTGATTTTTACAAGGCTCGATTCGATTGTCCCATGTCTGAAATTCTCAAAAGTCAGGATAGAAATGTCAAAGAAATAGTGTATCCCAAAGATGTGCCATGGAACATTGTTACACCTAGCAGAAAGTTGGTCGTGGTTCAGTTTAACCAATTTGATTGTGGAGGAATTGCTCTAAGTGCATGCGTTTCACACAAGATTGAAGATATGCATACATTTTATAAGTTTATGCATGATTGGGCTGCAATATCGCTCTGTTCTAACGTAAATATATGTCCTCAATTTATTGGATCGTCAGTTTTCCCACCTACAAATGAAGCTGTGAATGAACCACCTCGCGAACAATGTGTAACAAAGAGATTACTTTTTTCAAACCATGCATTGAAATCTCTCCTCCCAGGATCATCAGAAGTGAAAAATCCAACTCGGGTTGAAATACTGACAGCACTTCTTTATAAATGTAGTATGAGGGCGAATTCTAGTGGATTGTTCAAGCCATCAATGTTGTTTCAAACTATCAATTTAAGACCTATTATACCTTTGCCAGATAATACTCCTGGAAATTTCAGTTCTTCCCTTTTTGTACCAACATATACTGAAGAAGAAATGAAGTTATCAAGATTGGTTAGTGAGCTAAGGAAAGGAAAAGAAAAATATTTTGATGATTACAGAAAGTGTAAAGAAGGTCATCAAGATATGGTTTCTACAACAACGAGACCGTATCAAGAAATAAGGGCTCTGTTCAAGGACAATGATTTTGATCTTTATAGGTGTAGTAGTTTGGTGAATTATGGGTGGCATGGTTTAGACTTTGGATGGGGTATGCCTAATAGAGTAAGTATGGCAGACGTGAAACTGAGAAATATTTTCATGCTGTTCGATAACAACACAGAGGATCATGTAGAAGCACAGGTATCTTTTGACAAAGAAAGTAAAATGTCGGCGTTCTTACGAGAGATTGAGCAAGTTCTTG

>HnASAT2|HN_c61400_g1_i3

ATGGCTGCTCCAAGACTTGTTTTACTTTCCAAAAAGATCATTAAGCCTTCTTCTCCTACACCACTTTCACATAGAATTCAAAAGCTCTCTCTTATGGATCAAATGGGGACTCACTCCTACAGTCCATTTTCTTTCTTCTACCCCAAACAGGACACTGCAAGCTCGCCAGAACCAACAAAGGTATTCGAAATTCTTGAAAAATCCCTTTCCAAAGTCCTAACGGCTTATTATCCGTTTGCTGGACGCATAAGAGATAACTCCTTGATTGAATGTAATGACATGGGTGTCGAACTCTCTCACGTTCGAATTGATTGTCCAATGTCCACTATCTTCAATCACCCTCATACTGATATCGATAATTTGATCTTTCCAGACGATCCTTGGTTCCCATCCACGGAAAGTTTAGTCGTAGCTCAACTTAGTCATTTCAAATGTGGTGGCGTAGTGCTCGGTGCGTCCTTCTCCCACAAGGTCAGTGATGGGCTGAGTACGATTAAATTCCTACGGGACTATGCAATGGTTGCGCGTAATTCAGAAGCAAAACCTTCTCCCCTGTTTACTGGTGCGTCAATTTTTCAACCAATCAAATTTTCTTCATCGTCTCCCGTTATTGTTGATCCTCCTCGAAAACTAAATGCATCGAAAAGGTACCATTTTTCAACTTCCAAGCTAAAAGCTCTAAAGGCCTTTGTTTCAGCTGATTCCAAAAGCCAAATTCTTCCAACAACTGTGGAAGCTGTAACTGCATTCCTTTGCAAATGCGTTAACACGCCAACTTTTAAGCCATCATTATTGGTGCAGGCAGTTAACCTACGTGGAACAAATAATGATGCACTCGTTCCAGCAGACTTGTTCGGGAACGCCGTACTTCCTTTCGCTGTATCAGCAGCGAATAAGGAAGAGATAAATTTGCAAAGACTAGTTGGTGAGCTTAGAAAAGGAAAAGAGAAGATCCAAGATACGCTCAAATATGTTAAATCAGAAGAGTTGCTATGTTCAACGGTGTCTGAAATAGCTAGAGAGATGAACGAGCAGACCTCAAGCAACGATATGCCTATGTACAGATTTACTAGTTTAAGGAAATTCCCATTACATGACATAAATTTTGGATGGGGGAGGCCAAGAAGAGTGGATATGGCTACTTATCCGGTGAATATGTTTGTCTTGATGGATAACCTAAGTGGGGACGGAGTTGAAGTGCTCGTAAACTTGGAAGAAGGAGAGATGTCTGCATTTGAAAGTAACAACGAGCTTCATCAGTTTGCTTCTCCATTCTCGGGACTCTAG
