# Supplementary material for: Evolutionary routes to biochemical innovation revealed by integrative analysis of a plant-defense related specialized metabolic pathway
Source: eLife. 2017 Aug 30;6:e28468. doi: 10.7554/eLife.28468 (PMC5595436; doi:10.7554/eLife.28468)
Supplement: Supplementary file 4. [file elife-28468-supp4.docx]

***Supplementary File 4: Experimental conditions for VIGS experiments***

| # | Date started | Date extracted | # of weeks post-inoculation | Genes tested | Empty vector control | Conditions ^1^ |
| --- | --- | --- | --- | --- | --- | --- |
| 1 | June 8 | Aug 13 | 10-11 wks | SsASAT3 | TRV2 | 4 wk old seedlings used for inoculation in peat pellets. After 1 month in growth chamber 1, these were transferred to 2 Sure Mix+1/2 sand in chamber 2. |
| 2 | Aug 24 | Oct 21 | 8-9 wks | SsASAT1 | TRV2 | 3 wk old seedlings with 2 true leaves grown in growth chamber 1. After 1 month, these were transferred to 2 Sure Mix+1/2 sand in growth chamber 2. |
| 3 | Feb 21 | Apr 15 | 7-8 wks | SsASAT2  SsASAT5 | TRV2-LIC | 2 wk old seedlings with 2 true leaves grown in growth chamber 2 on RediEarth |
| 4 | Feb 21 | Mar 15 | 3-4 wks | SsASAT1, SsASAT2  SsASAT3, SsASAT5 | TRV2-LIC | 3 wk old seedlings with 4 true leaves grown in growth chamber 2 on RediEarth^2^ |
| 5 | Apr 7 | May 4 | 3-4 wks | SsASAT1, SsASAT2  SsASAT5 | TRV2-LIC | 3 wk old seedlings with 4 true leaves grown in growth chamber on RediEarth |
| 6 | Apr 15 | May 9 | 3-4 wks | SsASAT2, SsASAT5 | TRV2-LIC | 3 wk old seedlings with 4 true leaves grown in growth chamber 2 on RediEarth |
| 7 | July 9 | Sept 3 | 7-8 wks | SsASAT1, SsASAT2 | TRV2-LIC | 4 wk old seedlings used for inoculation in peat pellets. After 1 month in growth chamber 2, these were transferred to 2 Sure Mix+1/2 sand in the same chamber. |

^1^ *In all cases, growth chamber 1 conditions included 16 hours daylight, with day/night temperatures being ~22C. Growth chamber 2 had 16 hours daylight with 22/12 as day/night temperatures. Relative humidity in chamber 2 was constant at 50%.*

*^2^ These conditions seem to be the most optimal. Plants at this stage are more robust and can withstand Agro infiltration without significant negative effects. Final sampling can be completed relatively quickly, in just 3-4 weeks after infiltration. However, certain phenotypes (eg: SsASAT1 knockdown) are better visible under a different set of conditions.*
